# A Truncated Singleton NLR Causes Hybrid Necrosis in *Arabidopsis thaliana*

**DOI:** 10.1093/molbev/msaa245

**Published:** 2020-09-23

**Authors:** Ana Cristina Barragan, Maximilian Collenberg, Jinge Wang, Rachelle R Q Lee, Wei Yuan Cher, Fernando A Rabanal, Haim Ashkenazy, Detlef Weigel, Eunyoung Chae

**Affiliations:** 1 Department of Molecular Biology, Max Planck Institute for Developmental Biology, Tübingen, Germany; 2 Department of Biological Sciences, National University of Singapore, Singapore

**Keywords:** hybrid incompatibility, autoimmunity, singleton NLR, *DM10*, LRR–PL region, interchromosomal relocation

## Abstract

Hybrid necrosis in plants arises from conflict between divergent alleles of immunity genes contributed by different parents, resulting in autoimmunity. We investigate a severe hybrid necrosis case in *Arabidopsis thaliana*, where the hybrid does not develop past the cotyledon stage and dies 3 weeks after sowing. Massive transcriptional changes take place in the hybrid, including the upregulation of most NLR (nucleotide-binding site leucine-rich repeat) disease-resistance genes. This is due to an incompatible interaction between the singleton TIR-NLR gene *DANGEROUS MIX 10* (*DM10*), which was recently relocated from a larger NLR cluster, and an unlinked locus, *DANGEROUS MIX 11* (*DM11*). There are multiple *DM10* allelic variants in the global *A. thaliana* population, several of which have premature stop codons. One of these, which has a truncated LRR–PL (leucine-rich repeat [LRR]–post-LRR) region, corresponds to the *DM10* risk allele. The *DM10* locus and the adjacent genomic region in the risk allele carriers are highly differentiated from those in the nonrisk carriers in the global *A. thaliana* population, suggesting that this allele became geographically widespread only relatively recently. The *DM11* risk allele is much rarer and found only in two accessions from southwestern Spain—a region from which the *DM10* risk haplotype is absent—indicating that the ranges of *DM10* and *DM11* risk alleles may be nonoverlapping.

## Introduction

Hybrid necrosis, a common form of hybrid incompatibility in plants, is caused by conflicting elements of the plant immune system originating from different parental accessions. These pairwise deleterious epistatic interactions usually involve at least one nucleotide-binding site leucine-rich repeat (NLR) protein (Bomblies et al. 2007; Alcázar et al. 2009; Yamamoto et al. 2010; Chae et al. 2014; Sicard et al. 2015; Deng et al. 2019). NLRs function as intracellular immune receptors, similarly to NOD/CARD proteins in animals, and play a major role in plant innate immunity (Maekawa et al. 2011; Jones et al. 2016). The constant coevolutionary arms-race between plants and their pathogens has led to a high diversification of many elements of the plant immune system, including NLRs (Jones and Dangl 2006; Dodds and Rathjen 2010). Hybrid necrosis can be viewed as collateral damage resulting from, sometimes relatively minor, sequence differences between NLR alleles. This phenomenon can limit the possible NLR allele combinations found in an individual plant (Chae et al. 2014).

Plant NLRs are multidomain proteins usually composed of N-terminal Toll/interleukin-1 receptor/resistance protein (TIR), coiled-coil (CC), or RESISTANCE TO POWDERY MILDEW 8 (RPW8) domains, a central nucleotide-binding site (NBS), and C-terminal leucine-rich repeats (LRRs) (Meyers et al. 2003; Shao et al. 2016). The N-terminal domain is usually thought to be involved in signal transduction, whereas the NBS domain can act as a molecular ON/OFF switch (Bentham et al. 2017). The LRR domain is highly variable and consists of multiple repeats of 20–30 amino acid stretches that are often responsible for direct or indirect pathogen effector recognition as well as NLR autoinhibition (Ade et al. 2007; Krasileva et al. 2010; Steinbrenner et al. 2015). In addition, many TIR-NLRs carry a post-LRR (PL) domain, which is involved in pathogen effector recognition (Van Ghelder and Esmenjaud 2016; Martin et al. 2020).

Approximately half of all NLR genes in a given *Arabidopsis thaliana* accession are found in multigene clusters, which are unevenly distributed across the genome (Meyers et al. 2003; Van de Weyer et al. 2019). Tandem duplication events are common in NLR clusters, and duplicate genes are a major source of genetic variation, since they often experience relaxed selection and enable neofunctionalization (Ohno 1970; Force et al. 1999; Lynch and Conery 2000; Conant and Wolfe 2008). Sequence homogenization through intergenic exchange among cluster members is greatly reduced when an NLR gene is translocated away from its original cluster to an unlinked genomic region, thereby preserving its original function or potentially developing a new one (Baumgarten et al. 2003; Leister 2004). For NLRs, neofunctionalization of duplicated or translocated genes can expand the repertoire of pathogen effectors an individual plant is able to recognize (Botella et al. 1998; Michelmore and Meyers 1998; Holub 2001; Kim et al. 2017).

Genome-wide analysis of structural variation across eight high-quality *A. thaliana* genomes identified rearrangement hot spots coinciding with numerous multigene NLR clusters (Jiao and Schneeberger 2020), including the previously described *DANGEROUS MIX* (*DM*) loci, which are causal for hybrid necrosis (Bomblies et al. 2007; Chae et al. 2014). This raises the possibility that accelerated evolution associated with genomic rearrangements contribute to the generation of incompatibility alleles, pointing to genomic architecture as a driver of hybrid incompatibility. So far, over a dozen NLR loci with hybrid necrosis alleles are known from multiple plant species. Curiously, even though singletons account for about a quarter of NLRs in different species (Jacob et al. 2013), none of the causal NLR loci identified so far is a singleton NLR, here defined as a physical single-gene NLR and not to be confused with a functional singleton (Adachi et al. 2019). Most, but not all, well-characterized singleton NLRs, such as *RPM1* and *RPS2* in *A. thaliana*, show ancient balanced polymorphisms that maintain active and inactive alleles at intermediate frequencies in natural metapopulations (Caicedo et al. 1999; Stahl et al. 1999; Mauricio et al. 2003; Allen et al. 2004; MacQueen et al. 2016). Thus, with less functional diversity, and beneficial alleles often being relatively common, one would indeed expect that singleton NLRs are underrepresented among hybrid necrosis loci.

Here, we are investigating a case of severe hybrid necrosis, where hybrid plants do not develop past the cotyledon stage, become necrotic, and die 3 weeks after sowing. Extensive transcriptional changes occur in the hybrid, including the induction of most NLR genes. Through a combination of quantitative trait locus (QTL) analysis and genome-wide association studies (GWAS), we identified two new incompatibility loci, *DANGEROUS MIX 10* (*DM10*), a TIR-NLR on chromosome 5, and *DM11*, an unlinked locus on chromosome 1, as causal for incompatibility. *DM10* is an unusual hybrid incompatibility locus because it is a singleton NLR that arose after *A. thaliana* speciation through interchromosomal transposition from the *RLM1* cluster, which confers resistance to *Leptosphaeria maculans*, that causes blackleg disease in *Brassica* species (Staal et al. 2006; Guo et al. 2011). The causal allele has a premature stop codon that removes the C-terminal quarter of the protein, which includes part of the LRR–PL region, indicating that substantial NLR truncations can lead to hybrid incompatibility.

## Results

### A Particularly Severe Case of Hybrid Necrosis: Cdm-0×TueScha-9

**Fig. 1.**
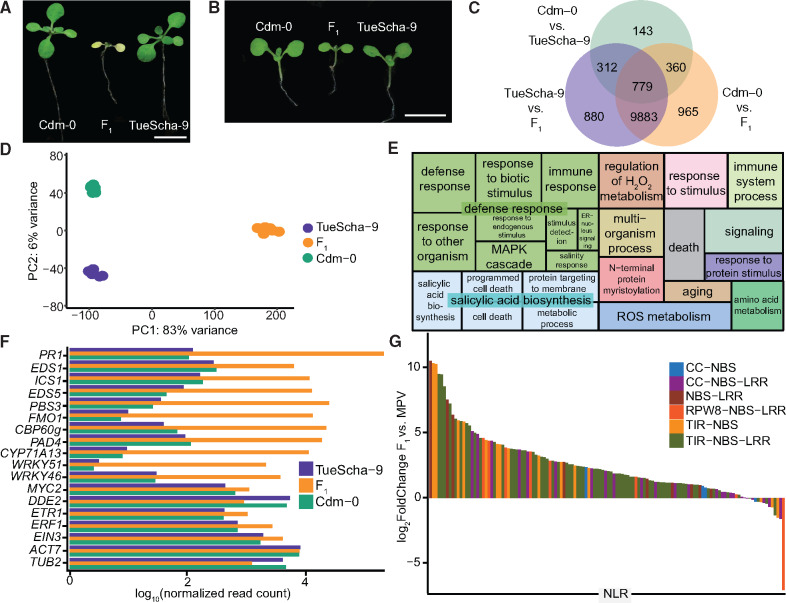
RNA-seq analysis of Cdm-0×TueScha-9 hybrids. (*A*) At 21 days, the Cdm-0×TueScha-9 F_1_ hybrid is necrotic. Plants were grown at 16 °C. Scale bar represents 1 cm. (*B*) Examples of a 10-day-old Cdm-0×TueScha-9 F_1_ hybrid and parental accessions harvested for RNA-seq. Plants were grown at 23 °C. Scale bar represents 1 cm. (*C*) Intersection of DEGs between the F_1_ hybrid and parents. (*D*) PCA of gene expression values. The main variance is between the F_1_ hybrid and parents. Each dot indicates one biological replicate, with six per genotype. (*E*) REVIGO Gene Ontology treemap. Size of the square represents −log_10_(*P* value) of each GO term. (*F*) Log_10_(normalized read count) of defense-related marker genes of the hybrid and the parents. (*G*) Differences in expression between the F_1_ hybrids and the midparental values (MPV) of NLR genes, with 128 significantly (|log_2_FoldChange|>1, *P*adj value <0.01) differentially expressed in at least one genotype comparison.

Eighty *A. thaliana* accessions have previously been intercrossed with the goal of identifying hybrid incompatibility hot spots (Chae et al. 2014). A particularly severe case was observed in the crosses between Cdm-0 and five other accessions: TueScha-9, Yeg-1, Bak-2, ICE21, and Leo-1. The F_1_ progeny of these two parents did not develop past the cotyledon stage, even at temperatures that suppress hybrid necrosis in most other cases (Chae et al. 2014), and severe necrosis developed during the second week after sowing, followed by complete withering in the third week (fig. 1*A*).

To obtain insights into the transcriptional changes in the hybrid, we performed RNA-seq on the parental accessions Cdm-0 and TueScha-9, as well as in F_1_ hybrid plants 10 days after germination, when the hybrid was already slightly stunted, but before there were visible signs of necrosis (fig. 1*B*). We observed massive transcriptional changes, in which around half of all 20,000 detectable genes (supplementary fig. S1*A*, Supplementary Material online) were differentially expressed in the hybrid when compared with either parent (fig. 1*C* and supplementary fig. S1*B* and table S1, Supplementary Material online). This represents one-third of the entire *A. thaliana* transcriptome (Klepikova et al. 2016). A principal component analysis (PCA) showed that most of the variance in gene expression is driven by the difference between the parents and the hybrid (PC1: 83%) (fig. 1*D*). In addition, we generated in silico hybrids (see Materials and Methods) and compared these with the biological F_1_ hybrids through a PCA. This confirmed that gene expression in the F_1_ hybrid is not an additive result of expression in the two parental accessions (supplementary fig. S1*C*, Supplementary Material online). Next, we carried out a Gene Ontology (GO) analysis using the top 1,000 differentially expressed genes (DEGs) from a comparison of the F_1_ hybrids and the midparental values (MPV) (supplementary table S2, Supplementary Material online). “Defense response” and “salicylic acid biosynthesis” were the categories with the highest number of DEGs in the hybrid versus MPV comparison (fig. 1*E* and supplementary table S2, Supplementary Material online).

Since the F_1_ hybrid displayed signs of an increased pathogen defense response, we analyzed the expression of a set of marker genes for defense-associated phytohormones such as jasmonic acid (JA), salicylic acid (SA), and ethylene (ET) (Papadopoulou et al. 2018), as well as early pathogen response genes induced by both cell surface receptors and NLRs (Ding et al. 2020) (supplementary table S3, Supplementary Material online). Genes involved in SA biosynthesis and signaling, such as *EDS1*, *ICS1*, *EDS5*, *PAD4*, *PBS3*, *CBP60*, and *FMO1*, were strongly overexpressed in F_1_ hybrid plants, in concordance with the GO analysis, as was the SA-induced camalexin biosynthesis gene *CYP71A13*. The expression of genes encoding transcription factors *WRKY46* and *WRKY51* and of the late immune response gene *PR1* was also increased in the hybrid (fig. 1*F* and supplementary table S3, Supplementary Material online). In contrast, the expression of genes required for JA-mediated resistance, such as *MYC2* or *DDE2*, or genes involved in ET signaling, such as *ETR1*, *ERF1*, and *EIN3*, changed to a lesser extent in the F_1_ hybrid, similar to control genes *ACT7* and *TUB2* (fig. 1*F* and supplementary table S3, Supplementary Material online).

Since an increase in NLR expression has been linked to autoimmunity (Stokes et al. 2002; Mackey et al. 2003; Palma et al. 2010; Lai and Eulgem 2018), and since some NLRs are upregulated when SA levels rise (Shirano et al. 2002; Yang and Hua 2004; Tan et al. 2007; Mohr et al. 2010; MacQueen and Bergelson 2016), we set out to investigate NLR expression levels in the hybrid. Out of a set of 166 NLRs found in the Col-0 genome, 150 were expressed in at least one of the three genotypes studied, and 128 were significantly (|log_2_FoldChange|>1, *P*adj value <0.01) differentially expressed in at least one genotype comparison (supplementary fig. S2*D* and table S4, Supplementary Material online). From these 128 NLRs, all but one were differentially expressed when comparing the hybrid with either parental accession (supplementary fig. S1*D*, Supplementary Material online). NLRs were mostly upregulated in the F_1_ hybrid: of the 95 NLRs with significant expression changes in the hybrid versus the MPV, all but three were overexpressed (fig. 1*G* and supplementary fig. S1*E* and table S1, Supplementary Material online). When the F_1_ hybrid was compared with the parents, the expression of individual NLRs largely followed the same pattern (supplementary fig. S1*E*–*G*, Supplementary Material online), this was not the case when comparing the two parents (supplementary fig. S1*H*, Supplementary Material online). The fraction of genes overexpressed in the F_1_ hybrid was similar for the different NLR classes as well as between singleton and clustered NLRs (fig. 1*G* and supplementary fig. S1*F*–*H* and table S3, Supplementary Material online).

### QTL Mapping of *DM10* and *DM11* in a Triple-Hybrid Cross

**Fig. 2.**
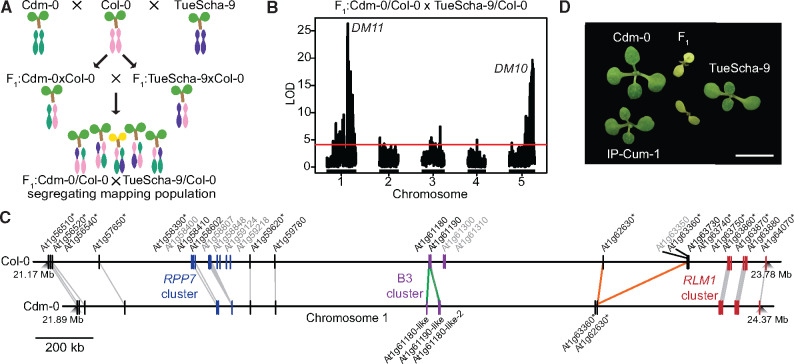
QTL mapping with a triple-hybrid cross. (*A*) Creation of a Cdm-0×TueScha-9 mapping population. (*B*) QTL analysis from polymorphic Cdm-0 and TueScha-9 markers. QTL peaks are found on chromosome 5 (23.35–24.45 Mb), *DM10*, and chromosome 1 (21.55–22.18 Mb), *DM11*. The horizontal lines indicate 0.05 significance threshold established with 1,000 permutations. (*C*) Comparison and distribution of candidate *DM11* NLR genes between At1g56510 and At1g64070 on chromosome 1. Gene IDs in gray are present in Col-0 but not in Cdm-0, gene duplications are marked in green and inversion events in orange. Asterisks indicate significant (|log_2_FoldChange|>1, *P*adj value <0.01) gene expression changes in the F_1_ hybrid when compared with the MPV. (*D*) Cdm-0×TueScha-9 and IP-Cum-1×TueScha-9 hybrids. Plants are 2 weeks old and were grown at 16 °C. Scale bar represents 1 cm.

Having found that a very large fraction of NLR genes is upregulated in the Cdm-0×TueScha-9 hybrid, we wondered whether hybrid necrosis in this case was due to global NLR regulators (Li et al. 2009; Zhai et al. 2011; Shivaprasad et al. 2012; Gloggnitzer et al. 2014; Sicard et al. 2015), or to NLRs, as in other hybrid necrosis cases. We therefore proceeded to map the underlying causal loci via QTL analysis. Since the F_1_ hybrid seedlings died very young, we could not directly generate a segregating F_2_ mapping population (Bomblies et al. 2007; Chae et al. 2014; Barragan et al. 2019). Instead, we designed a triple-hybrid cross (Cooper et al. 2019) and first generated two sets of heterozygous plants by crossing Cdm-0 and TueScha-9 separately to a third, innocuous background, the Col-0 reference accession. We then intercrossed these Cdm-0/Col-0 and TueScha-9/Col-0 plants (fig. 2*A*). In the resulting pseudo-F_2_ generation, we collected both normal and necrotic plants and individually genotyped them by RAD-seq (Rowan et al. 2017). For QTL mapping, we focused on polymorphic markers between Cdm-0 and TueScha-9, including markers overlapping with the Col-0 reference (fig. 2*B*), and also analyzed polymorphic markers for each accession independently (supplementary fig. S2*A* and *B*, Supplementary Material online). We identified two genomic regions that interact epistatically to cause the severe hybrid necrosis phenotype. We called the QTL on chromosome 5 (23.35–24.45 Mb) *DM10*, and the QTL on chromosome 1 (21.55–22.18 Mb) *DM11*. Both intervals contain NLRs but no clear candidates for global NLR regulators, so we chose to focus on NLR genes. In the *DM10* mapping interval, one NLR is present, At5g58120, whereas the *DM11* interval was NLR-rich and encompassed ten NLRs in Col-0 (supplementary table S5, Supplementary Material online). Loci in the interval include the highly polymorphic *RPP7* cluster of CC-NLR genes (McDowell et al. 2000; Guo et al. 2011; Li et al. 2020), as well as two CC-NLR singleton genes, *CW9* (At1g59620) and At1g59780 (Meyers et al. 2003). To identify potential differences between Col-0 and Cdm-0 in the *DM11* interval, we generated a PacBio long read-based genome assembly of this accession (supplementary table S6, Supplementary Material online). Notably, most chromosome arms were assembled in single contigs, including the long arm of chromosome 1, where the *DM11* mapping interval is located (supplementary fig. S3, Supplementary Material online). Since at the time the full Cdm-0 annotation was not yet available, we manually annotated homologs of NLR genes corresponding to the genomic region that spans from At1g56510 to At1g64070 in Col-0, which includes the *DM11* mapping interval as well as neighboring NLRs. Like Col-0, Cdm-0 carries groups of both clustered and singleton NLRs, adding up to a total of 21 NLRs, compared with 28 NLRs in Col-0 (fig. 2*C* and supplementary table S5, Supplementary Material online).

To pinpoint *DM11* candidate genes, we sought to identify additional accessions that had similar alleles as Cdm-0 at *DM11* candidate loci by creating neighbor-joining (NJ) trees (supplementary fig. S2*C*, Supplementary Material online) and PCA plots (supplementary fig. S2*D* and table S7, Supplementary Material online), using sequences from the 1001 Genomes Project (1001 Genomes Consortium 2016). IP-Cum-1 was the accession most similar to Cdm-0 for the whole *DM11* mapping interval, and when we crossed it to TueScha-9, Cdm-0×TueScha-9-like hybrid necrosis was observed (fig. 2*D*). Eleven other accessions that were less closely related to Cdm-0 in this genomic interval did not produce necrotic F_1_ hybrids (supplementary table S7, Supplementary Material online). Because accessions Istisu-1 and ICE134, like Cdm-0, lack a transposable element that is present in most *RPP7* (At1g58602) alleles (Tsuchiya and Eulgem 2013), we also crossed these two accessions to TueScha-9, but no hybrid necrosis was observed (supplementary table S7, Supplementary Material online). Artificial miRNAs (amiRNAs) (Schwab et al. 2006) targeting different members of the *RPP7* cluster were previously designed to perform rescue experiments for other cases of hybrid necrosis (Chae et al. 2014; Barragan et al. 2019); although predicted to target all members of the Cdm-0 *RPP7* cluster, neither these nor amiRNAs targeting *CW9*^Cdm-0^ or At1g59780^Cdm-0^ suppressed hybrid necrosis (supplementary table S8, Supplementary Material online). Lastly, a genomic *CW9*^Cdm-0^ fragment was unable to induce hybrid necrosis when introduced into TueScha-9 (supplementary table S18, Supplementary Material online).

Being aware that the precision of QTL mapping in NLR-rich regions can be affected by structural variation, we also tested NLRs adjacent to the *DM11* mapping interval. The *RLM1* cluster is highly similar among Cdm-0 and IP-Cum-1, both of which carry the causal *DM11* allele in addition, some cluster members show an increased expression in the F_1_ hybrid, which is sometimes the case for causal NLRs (Bomblies et al. 2007) (supplementary fig. S2*E*, Supplementary Material online). We therefore tested six of the seven *RLM1* cluster members via *Nicotiana benthamiana* coexpression with *DM10*^TueScha-9^ (see fig. 4 for cloning of causal *DM10* allele), but none induced a hypersensitive response (HR) (supplementary table S5, Supplementary Material online). Six accessions with a similar *RLM1* locus to that of Cdm-0 and IP-Cum-1 were crossed with TueScha-9, but no necrosis was observed (supplementary table S7, Supplementary Material online). Finally, because At1g57650 was strongly upregulated among *DM11* NLR candidate genes, we tested it with *DM10*^TueScha-9^ in *N. benthamiana*, but again no HR was observed (supplementary fig. S2*E* and tables S4 and S5, Supplementary Material online). This may indicate that *DM11* is either an NLR that was not tested, or not an NLR at all. Other *DM11* candidates may include any of the genes in this interval that encode proteins that are not annotated as NLRs but have a TIR or LRR domain (supplementary tables S9–S11, Supplementary Material online). Note that some Col-0 NLRs that had no homologs in the interval from At1g56510 to At1g64070 in Cdm-0 attracted nonspecific RNA-seq reads, most likely because there are homologs elsewhere in the Cdm-0 genome (supplementary table S5, Supplementary Material online).

### Fine-Mapping of *DM10* Using Genome-Wide Association Studies

**Fig. 3.**
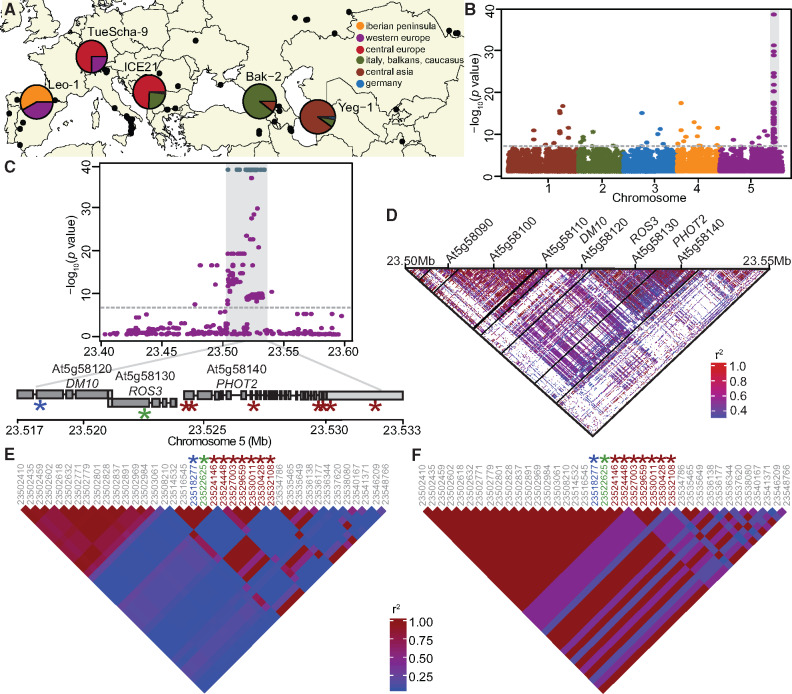
GWAS of hybrid necrosis in 80 accessions. (*A*) Map of 80 accessions (black dots), with the five risk accessions colored according to 1001 Genomes admixture groups (1001 Genomes Consortium 2016). (*B*) Manhattan plot for association of necrosis in Cdm-0 hybrid progeny when selfed and crossed to 79 other accessions. The significance threshold (Bonferroni correction, 5% family-wise error) is indicated as a horizontal dotted line (same in *C*). (*C*) Close-up of the region highly associated with hybrid necrosis; SNPs with a 1:1 association marked in teal. Asterisks indicate such 1:1 associations in At5g58120, *ROS3*, and *PHOT2*; see also (*E*) and (*F*). SNP positions are given in supplementary table S13, Supplementary Material online. (*D*) Linkage disequilibrium (LD) across a 50-kb region in chromosome 5. Strong linkage is observed from At5g58090 to At5g58140. (*E*) LD across the same 50-kb region as in (*D*), with a subset of markers from 80 accessions crossed to Cdm-0. Asterisks indicate markers highlighted in (*C*). (*F*) LD across a 50-kb region with the same markers as in (*E*), but for the five risk accessions only. Higher LD is seen here than in (*E*).

In the original collection of 6,409 crosses among 80 accessions (Chae et al. 2014), four accessions in addition to TueScha-9 produced severe hybrid necrosis when crossed to Cdm-0: Yeg-1, Bak-2, ICE21, and Leo-1. Together with TueScha-9, these represent much of the Eurasian range of the species, both geographically and genetically; six of the nine previously identified admixture groups (1001 Genomes Consortium 2016) are present in these five risk accessions (fig. 3*A* and supplementary table S12, Supplementary Material online). Given the diversity of the five incompatible accessions, and knowing that most, but not all, causal genes for hybrid incompatibility are NLRs, we attempted to narrow down causal *DM10* candidate genes by GWAS, with Cdm-0-dependent F_1_ necrosis as a binary trait (Grimm et al. 2017). We discovered a remarkably high association between this phenotype and several closely linked markers on the bottom of chromosome 5, with corrected *P* values as low as 10^−38^. In addition, 79 SNPs showed a one-to-one association with the necrotic phenotype, resulting in −log_10_  *P* values of 0 (fig. 3*B* and supplementary table S13, Supplementary Material online). The markers with the strongest associations tagged three loci: At5g58120, encoding a TIR-NLR without known function, *ROS3* (At5g58130), encoding an enzyme involved in DNA demethylation (Zheng et al. 2008), and *PHOT2* (At5g58140), encoding a blue light receptor that mediates phototropism (Harada et al. 2003) (fig. 3*C* and supplementary table S13, Supplementary Material online). These three loci are genetically similar among the five risk accessions, yet differentiated from the other 75 accessions used for GWAS (supplementary fig. S4*A*–*C*, Supplementary Material online). Looking at linkage among loci in this genomic region, we could see that, when taking all 80 accessions into account, six loci (At5g58090–Atg58140) belong to one large linkage block, in which *ROS3* and *PHOT2* are under tight linkage and the TIR-NLR At5g58120 constitutes a separate linkage block (fig. 3*D*). Notably, in the five accessions causing hybrid incompatibility, stronger linkage is observed in this region than that seen among the same markers from all 80 accessions (fig. 3*E* and *F*). In the risk accessions, At5g58120, *ROS3*, and the proximal part of *PHOT2* form one linkage block, whereas SNPs located in the distal half of *PHOT2* are found in a separate linkage block, rendering At5g58120 and *ROS3* as primary candidates for causality in hybrid necrosis (fig. 3*F*).

### *DM10*, a Singleton TIR-NLR, as Cause of Severe Hybrid Necrosis

**Fig. 4.**
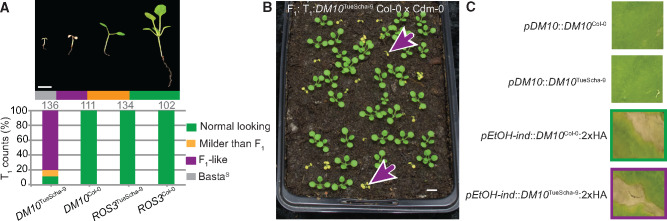
Experimental identification of *DM10*. (*A*) Recapitulation of hybrid necrosis in 25-day-old Cdm-0 T_1_ plants transformed with the indicated genomic fragment from TueScha-9 or Col-0. Representative phenotype and total number of T_1_ plants examined given on top. Plants were grown at 16 °C. Scale bar represents 1 cm. (*B*) The same *DM10*^TueScha-9^ genomic fragment as in (*A*) was introduced into Col-0, and T_1_ plants were crossed to Cdm-0. The F_1_ hybrid phenotype was recapitulated (magenta arrows). Plants were 18 days old and grown at 16 °C. Scale bar represents 1 cm. (*C*) Infiltration of *Nicotiana benthamiana* leaves with the indicated constructs. Overexpression of either *DM10*^TueScha-9^ or *DM10*^Col-0^ under an EtOH-inducible promoter (*pEtOH-ind*) triggered cell death, whereas expression from their native promoter (*pDM10*) did not. Images were taken 7 days after infection.

Having candidate genes for *DM10*, we next sought to experimentally test their causality for severe hybrid necrosis. Genomic fragments of the TIR-NLR At5g58120 and *ROS3*, from both Col-0 and TueScha-9, were introduced into Cdm-0 plants. A 4.8-kb genomic fragment containing At5g58120^TueScha-9^ recapitulated the Cdm-0×TueScha-9 hybrid necrosis phenotype (fig. 4*A* and supplementary table S14, Supplementary Material online). At5g58120 is henceforth called *DM10*. When *DM10*^TueScha-9^ was introduced into a Col-0 background and the resulting T_1_ plants were subsequently crossed to Cdm-0, we also observed the hybrid incompatibility phenotype in the F_1_ progeny (fig. 4*B*). *DM10*^Col-0^, *ROS3*^TueScha-9^, and *ROS3*^Col-0^ did not produce any necrosis when introduced into a Cdm-0 background (supplementary table S14, Supplementary Material online). We also observed that, when infiltrated in *N. benthamiana* leaves and overexpressed under an EtOH-inducible promoter, both *DM10*^Col-0^ and *DM10*^TueScha-9^ were able to trigger cell death, which was not the case when *DM10*^Col-0^ and *DM10*^TueScha-9^ were expressed under the control of their native promoters (fig. 4*C*). The cell death-triggering activities under forced overexpression in the heterologous *N. benthamiana* system indicate that these NLRs are competent in immune signaling and, in the case of DM10^TueScha-9^, this is not abolished by its substantial truncation.

### Prevalence and Genetic Differentiation of the *DM10* Risk Allele in the Global *A. thaliana* Population

**Fig. 5.**
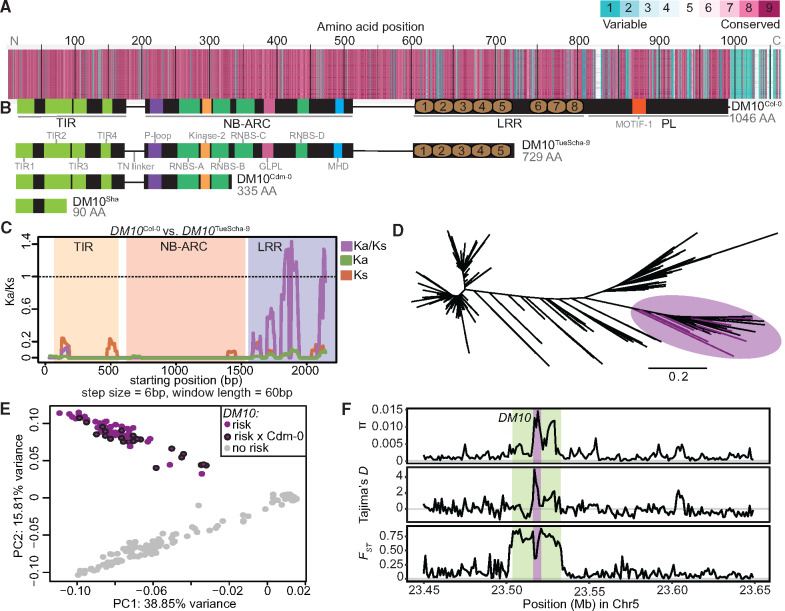
*DM10* natural variation. (*A*) Amino acid alignment of 73 DM10 proteins color-coded by its conservation score (Armon et al. 2001). (*B*) Comparison between different DM10 proteins (aligned with *A*). (*C*) Ka/Ks ratios between *DM10*^Col-0^ and *DM10*^TueScha-9^. (*D*) NJ tree of a region including *DM10*, *ROS3*, and *PHOT2* sequences from the 1001 Genomes Project (1001 Genomes Consortium 2016). Branch lengths in nucleotide substitutions are indicated. Accessions carrying the *DM10* risk alleles group together in a branch (magenta), risk accessions crossed to Cdm-0 are highlighted. (*E*) PCA. Accessions carrying the predicted *DM10* risk (magenta) versus nonrisk (gray) alleles are clearly separated in PC2. Risk accessions crossed to Cdm-0 are outlined in black. (*F*) *π*, Tajima’s *D* and *F*_ST_ for *DM10* (magenta), the *DM10* linkage block comprising At5g58090–Atg58140 (green) and surrounding genomic regions.

To study natural variation across different *DM10* alleles, 73 alleles belonging to accessions originating from across *A. thaliana*’s native range were extracted from preliminary short- and long-read genome assemblies available in-house (supplementary fig. S5*A* and table S15, Supplementary Material online). A maximum-likelihood tree of these alleles showed that there are multiple well-supported DM10 clades (supplementary fig. S5*B*, Supplementary Material online), and that variation between DM10 proteins was most prevalent at the C-terminal end (fig. 5*A* and supplementary fig. S5*C* and table S16, Supplementary Material online). Ten alleles were predicted to produce proteins truncated at three different points. Four accessions, including TueScha-9, the original *DM10* risk allele carrier, share the same stop codon (fig. 5*B* and supplementary fig. S5*B*, Supplementary Material online), removing three LRRs and the PL. Three short motifs have been previously recognized as being conserved in PLs of different NLRs (Van Ghelder and Esmenjaud 2016) (fig. 5*B* and supplementary fig. S5*C*, Supplementary Material online). The DM10 PL has a variant of the first of these motifs in a degenerate form. Five accessions had shorter, 335 amino acid long DM10 proteins; in these, the NBS domain was truncated, lacking motifs which are important regulators of NLR activity (Bendahmane et al. 2002; Sueldo et al. 2015; Bentham et al. 2017) (fig. 5*B* and supplementary fig. S5*C*, Supplementary Material online). These five accessions carrying short *DM10* alleles included Cdm-0 and IP-Cum-1, which also carry *DM11* risk alleles. This implies that the short Cdm-0-like *DM10* variants do not interact with *DM11* to produce hybrid necrosis. The shortest predicted DM10 protein, found in the Sha accession, is only 90 amino acids long and is truncated midway through the TIR-2 motif (fig. 5*B* and supplementary fig. S5*C*, Supplementary Material online). There are full-length DM10 alleles without any nonsynonymous differences to DM10^Cdm-0^ and DM10^Sha^, and that are distinguished from these only by truncation. Similarly, the full-length DM10^Col-0^ and the truncated DM10^TueScha-9^ proteins differ only at 3% of shared sites, which is low for within-species variation among NLR alleles (Van de Weyer et al. 2019). Furthermore, not only the coding sequence but also the sequence after the premature stop codon of the short *DM10* alleles, is highly similar to that of full-length alleles. This lack of signs of pseudogenization suggests that the truncations occurred relatively recently. As is typical for NLRs (Mondragón-Palomino et al. 2002; Ruggieri et al. 2014), Ka/Ks values >1 were found in the LRR domain when comparing DM10^Col-0^ and DM10^TueScha-9^ (fig. 5*C*). In contrast, DM10^Col-0^ and DM10^Lerik1-3^, which are both full-length DM10 proteins but from different clades, are more differentiated in TIR and NB-ARC domains, although Ka/Ks values >1 are also restricted to the LRR domain (supplementary fig. S5*D*, Supplementary Material online).

Next, to assess how common the *DM10* risk allele is in the global *A. thaliana* population, we again turned to the 1001 Genomes collection (1001 Genomes Consortium 2016). Since *DM10*, *ROS3*, and the proximal part of *PHOT2* were strongly linked in accessions carrying the *DM10* risk allele, we focused on this region, which contained 785 SNPs. In an NJ tree, all five confirmed *DM10* risk allele carriers were found in the same branch, which included 95 other accessions (fig. 5*D* and supplementary table S17, Supplementary Material online). In a PCA of this region, these 100 accessions were clearly separated from the rest (fig. 5*E*), which was not the case in a whole-genome PCA (supplementary fig. S5*E*, Supplementary Material online), indicating that population structure is not the main driving force separating risk from nonrisk allele carriers. To experimentally confirm that sequence was predictive of interaction with the *DM11* risk allele, 25 of the 100 accessions were crossed to Cdm-0 (supplementary fig. S5*B* and table S17, Supplementary Material online). All but IP-Alm-0 produced hybrid necrosis. Notably, whereas DM10 from IP-Alm-0 is 99.2% identical with DM10^TueScha-9^, it does not have the LRR truncation (supplementary fig. S5*B*, Supplementary Material online). This implies that the truncation in *DM10* risk alleles is likely responsible for incompatibility, and not individual amino acid changes. Ten random accessions not predicted to carry the *DM10* risk allele were crossed to Cdm-0 as a control; as expected, none produced hybrid necrosis (supplementary table S17, Supplementary Material online). Similarly, we investigated how common the other two DM10 truncations are in the global *A. thaliana* population. The shortest Sha-like *DM10* allele was found in 29 accessions, whereas the Cdm-0-like truncation is more common, although not as common as the *DM10* risk allele, and was found in 67 accessions (supplementary table S17, Supplementary Material online).

In a 200-kb region around *DM10*, nucleotide diversity (*π*) was highest, up to 0.015, in the distal half of *DM10*, encoding the more polymorphic LRR domain (fig. 5*F*). However, in comparison with other TIR-NLRs present in most or all accessions, overall *DM10* nucleotide diversity was not uncommon (Van de Weyer et al. 2019). Values for Tajima’s *D* reached 4.8 in the proximal half of *DM10*, hinting at multiple *DM10* alleles being prevalent at intermediate frequencies in the global *A. thaliana* population, as is often the case for NLRs (Caicedo et al. 1999; Stahl et al. 1999; Bakker et al. 2006; Karasov et al. 2014). Lastly, the fixation index (*F*_ST_) between 98 accessions with predicted *DM10* risk alleles (excluding IP-Alm-0 and RAD-21, which did not have truncated LRR domains) and 1,037 nonrisk allele carrying accessions, peaked at 0.88 across the *DM10* linkage block (figs. 3*D*–*F* and 5*F*). This was the only peak detected both across the entire chromosome 5 (supplementary fig. S5*F*, Supplementary Material online) and the whole genome. Inside this block, a drop in *F*_ST_ is seen over the proximal half of *DM10*, which is consistent with this region being similar between risk and some nonrisk alleles (fig. 5*C*).

Taken together, these results show that there are multiple *DM10* alleles in the global *A. thaliana* population, three of which are predicted to result in truncated proteins due to the presence of premature stop codons, one of which is the *DM10* risk allele. Notably, the *DM10* risk allele is not only relatively common and genetically differentiated in our GWAS population but also in the global *A. thaliana* population.

### No Documented Co-occurrence of *DM10* and *DM11* Risk Alleles in the Global *A. thaliana* Population

**Fig. 6.**
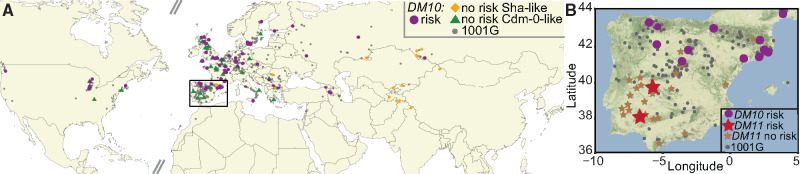
Geographical distribution of *DM10* and *DM11* alleles. (*A*) Geographical distribution of 1001 Genomes Project accessions (1001 Genomes Consortium 2016) carrying the *DM10* risk (magenta), nonrisk (gray) alleles, Sha-like nonrisk (orange), and Cdm-0-like nonrisk (green) alleles. Rectangle zooms into the region shown in (*B*). (*B*) Distribution of 1001 Genomes Project accessions (gray) in Spain and Portugal, carrying the *DM10* (magenta) and *DM11* (red) risk alleles, as well as accessions carrying *DM11* nonrisk alleles (brown) which were crossed to TueScha-9.

Looking at the geographical distribution of accessions carrying different *DM10* alleles with premature stop codons, we observed that both the Cdm-0-like *DM10* allele as well as the risk *DM10* allele were found at similar densities throughout *A. thaliana*’s native range, whereas the Sha-like *DM10* allele was mainly found toward the eastern part of the species’ distribution (fig. 6*A* and supplementary table S17, Supplementary Material online). In the case of the *DM10* risk allele, the one exception to where this allele was found, was the southwestern part of Spain and Portugal, even though *A. thaliana* has been heavily sampled in this region (1001 Genomes Consortium 2016). The fact that the only two *DM11* risk carriers identified so far, Cdm-0 and IP-Cum-1, are found in southwestern Spain may indicate that the *DM10* and *DM11* risk alleles do not geographically co-occur (fig. 6*B*).

To provide additional support for this assertion, we first attempted to identify more *DM11* risk carriers in Spain and Portugal. We crossed TueScha-9, a *DM10* risk allele carrier, to 24 accessions from these two countries, which were found at different geographical distances from the two *DM11* risk carriers Cdm-0 and IP-Cum-1, as well as from accessions carrying the *DM10* risk allele (supplementary table S7, Supplementary Material online). No hybrid necrosis was observed in any of the resulting F_1_ progeny (fig. 6*B* and supplementary table S7, Supplementary Material online). This, together with our aforementioned attempts to find additional *DM11* carriers among accessions that are closely related in the *DM11* genomic region to Cdm-0 and IP-Cum-1, indicates that the *DM11* risk allele is rare and potentially only found in southwestern Spain, a region where the *DM10* risk allele appears to be absent.

### Origin of the *DM10* NLR Singleton Locus through a Recent Interchromosomal Relocation Event Out of the *RLM1* Cluster

**Fig. 7.**
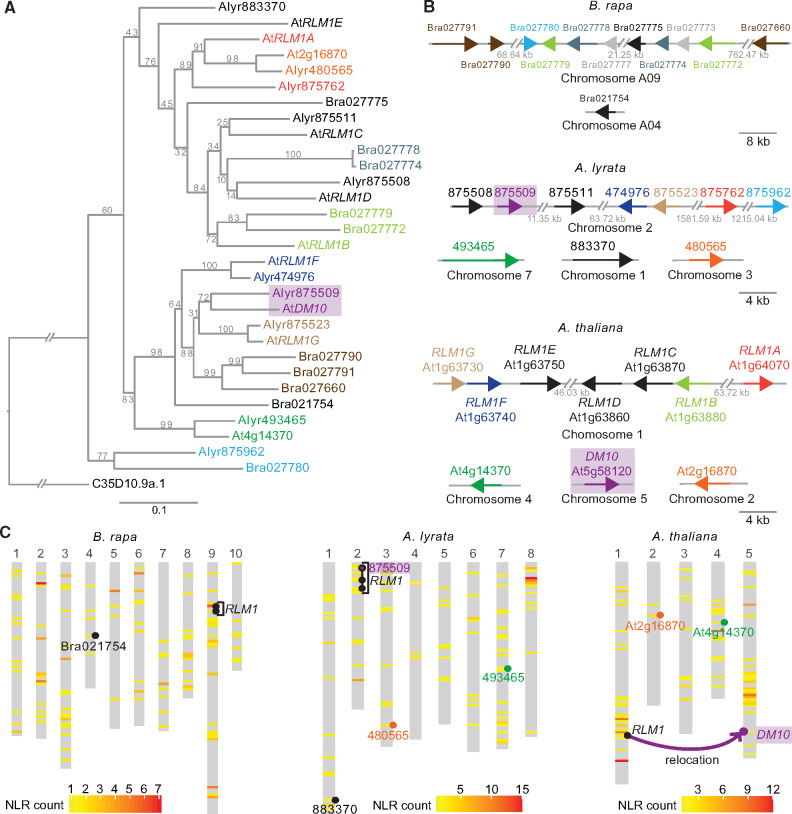
*RLM1* locus in *Brassica rapa*, *Arabidopsis lyrata*, and *Arabidopsis thaliana* reference genomes. (*A*) Maximum-likelihood tree of the NBS domain (CDS) of *RLM1* cluster members from *B. rapa*, *A. lyrata*, and *A. thaliana*, with the NBS domain of *Caenorhabditis elegans* CED-4 as outgroup (C35D10.9a.1). About 1,000 bootstrap replicates were performed, values are shown on each branch. Branch lengths in nucleotide substitutions are indicated. The same color was chosen for genes in neighboring branches with bootstrapping values >70. Diagonal lines indicate a gap in the tree branches. (*B*) *RLM1* cluster members and their homologs in *B. rapa*, *A. lyrata*, and *A. thaliana*. Color-coding the same as in (*A*), genes in gray are truncated, arrows represent size of NLR loci. Diagonal lines indicate a positional gap along the chromosome, the length of the gap is indicated. (*C*) Heatmaps of NLR densities across the three genomes. Window sizes were calculated by dividing the length of the longest chromosome by 100. *RLM1* cluster and closely related singletons indicated. *DM10* in *A. thaliana* and its homolog in *A. lyrata*, 875509 are highlighted in magenta.

In the *A. thaliana* Col-0 reference genome, we identified nine NLR genes closely related to *DM10*. Seven of these make up the *RLM1* cluster on chromosome 1, and two others, At2g16870, At4g14370 are dispersed singletons. In the related species *A. lyrata* and *Brassica rapa*, we identified a further 20 *DM10/RLM1* homologs (fig. 7*A*). As in *A. thaliana*, the cluster homologous to the *A. thaliana RLM1* cluster in these two species (not to be confused with the *RLM1* locus that provides resistance to blackleg disease in *Brassica* [Delourme et al. 2008; Fu et al. 2019]) underwent within-species duplication and inversion events (fig. 7*B*). Most *RLM1* members from *A. thaliana* have a clear one-to-one homolog in *A. lyrata*, so the expansion of the *RLM1* cluster must have occurred before the two species diverged (Beilstein et al. 2010). The *A. lyrata* homologs of At2g16870 and At4g14370, 480565 and 493465, are found in a different chromosome than the main *RLM1* cluster (fig. 7*C*). This is not the case for the *DM10* homolog from *A. lyrata*, 875509, which is located inside the main *RLM1* cluster (fig. 7*C*). This indicates that *DM10* was relocated away from the main *RLM1* cluster to another chromosome and that this occurred after *A. lyrata* and *A. thaliana* diverged.

## Discussion

Over ten causal genes for hybrid necrosis have been identified in *A. thaliana* and other plants (Krüger et al. 2002; Bomblies et al. 2007; Alcázar et al. 2009; Jeuken et al. 2009; Yamamoto et al. 2010; Chae et al. 2014; Chen et al. 2014; Todesco et al. 2014; Sicard et al. 2015; Deng et al. 2019; Sandstedt et al. 2020). In many instances, at least one of the two causal genes is an NLR, which is also the case for the Cdm-0×TueScha-9 incompatibility. What makes this case particularly interesting is the extreme severity of hybrid necrosis, the transcriptional hyperinduction of NLR genes, and the causality of a truncated singleton NLR, *DM10*, which was recently relocated from a larger NLR cluster.

As with normal immune responses and autoimmune syndromes, the expression of hybrid necrosis is typically temperature-dependent, and hybrid necrosis in *A. thaliana* can usually be completely suppressed when grown >23 °C (Bomblies et al. 2007; Alcázar et al. 2009; Chae et al. 2014; Todesco et al. 2014; Świadek et al. 2017). In contrast, the extreme autoimmune response in Cdm-0×TueScha-9 F_1_ seedlings cannot be rescued even by growing these hybrids at 28 °C (Chae et al. 2014). In other necrotic hybrids, noncausal NLRs have been reported to be differentially expressed between hybrids and their parents (Bomblies et al. 2007; Atanasov et al. 2018), but the NLR induction seen in Cdm-0×TueScha-9 is clearly the most extreme. For example, in the F_1_ progeny, 128 of 150 expressed NLRs are differentially expressed in at least one genotype comparison, with almost all being overexpressed. When we reanalyzed published data from another, relatively strong hybrid necrosis case, we found 104 out of 166 NLR genes to be differentially expressed, yet both extreme as well as the mean of overexpression was lower than in Cdm-0×TueScha-9 hybrids (supplementary fig. S6 and tables S1–S4, Supplementary Material online). In addition, the specific NLR genes that are overexpressed differ in both cases, indicating that specific NLRs respond differently depending on their genetic background (supplementary fig. S6 and table S4, Supplementary Material online). Simultaneous upregulation of several NLR genes has been observed after exposure to biotic (Zipfel et al. 2004; Tan et al. 2007; Ribot et al. 2008; Mohr et al. 2010; Yu et al. 2013; Sohn et al. 2014; Chen et al. 2016; Mine et al. 2018; Steuernagel et al. 2020) and abiotic stresses (MacQueen and Bergelson 2016), but not to the extent seen in Cdm-0×TueScha-9 hybrids. Given that elevated NLR expression levels can trigger cell death (Stokes et al. 2002; Mackey et al. 2003; Palma et al. 2010; Lai and Eulgem 2018), we expect that widespread NLR hyperinduction is a significant contributor to the strongly necrotic phenotype of Cdm-0×TueScha-9 F_1_ hybrids.

NLR transcript levels are tightly controlled through a variety of regulatory mechanisms (Lai and Eulgem 2018), and large-scale upregulation of NLRs could possibly require multiple pathways. We found WRKY transcription factors to be overexpressed in the hybrids; these proteins bind to W box motifs enriched in the promoters of multiple members of the plant immune system, including NLRs, and can induce widespread NLR expression, enhancing basal immunity (Eulgem and Somssich 2007; Pandey and Somssich 2009; Mohr et al. 2010). Two other mechanisms known to affect a broad set of NLRs are the miRNA-dependent phasiRNA production (Zhai et al. 2011; Li et al. 2012; Shivaprasad et al. 2012; Xia et al. 2015) as well as nonsense-mediated decay (Gloggnitzer et al. 2014), both of which help to dampen NLR gene expression in the absence of pathogen threats. Repression is attenuated after an incoming pathogen is detected by the plant, enabling global NLR levels to increase (Lai and Eulgem 2018). Although we have no direct evidence for transcription factors, small RNAs, or nonsense-mediated decay as contributors to aberrant NLR expression in the Cdm-0×TueScha-9 hybrid, this exceptional hybrid necrosis case may present a good tool for comparing NLR regulation under pathogen attack with strong autoimmunity.

We found 17% of DM10 proteins encoded in a global set of *A. thaliana* accessions to be truncated in either their TIR, NBS, or LRR domain. Similar to several full-length variants, the alleles for all three truncated proteins have intermediate frequencies and are relatively wide spread, suggesting that they are actively maintained in the global population by balancing selection. The most common of the three truncation alleles is the *DM10* risk version, which lacks three of the eight LRRs and the PL domain, and which shows evidence for its LRR domain being under diversifying selection. Although the TIR domain alone can induce cell death (Swiderski et al. 2009; Bernoux et al. 2011), a complete NBS domain is essential in many instances (Dinesh-Kumar et al. 2000; Dodds et al. 2001; Bendahmane et al. 2002; Tameling et al. 2002; Williams et al. 2011; Steinbrenner et al. 2015; Sueldo et al. 2015; Wang et al. 2015; Bernoux et al. 2016). NLRs lacking the NBS or LRR domain are not only known to retain the ability to cause cell death but there are cases where truncated NLRs are bona fide resistance genes (Nishimura et al. 2017; Roth et al. 2017; Marchal et al. 2018). Conversely, other proteins, including at least one full-length NLR, can induce cell death through activation of naturally occurring truncated NLRs (Zhao et al. 2015; Zhang, Wang, et al. 2017). In the case of *DM10*, we do not know whether only the full-length variants or the truncated variants, or both, are functional, and if they confer resistance to unknown pathogens, even though their prevalence and geographical distribution suggest so. Alternatively, the “less is more” hypothesis (Olson 1999) may explain the wide prevalence of truncated *DM10* alleles even if these are nonfunctional. Minor mutations in these alleles could readily remove the premature stop codons, making them “nearly functional” alleles that could act as easily activable functional reservoirs, as previously discussed for *RPM1* (Rose et al. 2012). The particular length of the risk DM10^TueScha-9^ protein combines the autoactive tendencies associated with the partial loss of the LRR–PL domain (Qi et al. 2018) with what appear to be functional TIR and NBS domains.

Because NLR allelic diversity is often not easily captured by short read-based resequencing (Van de Weyer et al. 2019), we still do not have a good grasp on whether NLR alleles in general, and specifically beneficial alleles, spread through the population more quickly than other adaptive alleles. The Iberian peninsula is a center of *A. thaliana* genetic diversity, with strong geographical structure across a north–south latitudinal gradient (Picó et al. 2008; Brennan et al. 2014). We observed a lack of co-occurrence between *DM11* risk alleles, restricted to southwestern Spain, and *DM10* risk alleles, restricted to the northern half of Spain (1001 Genomes Consortium 2016). Absence of co-occurrence between risk alleles may partly be the result of population structure: two geographical barriers potentially reducing gene flow, the Tagus river and the Central System mountains, divide populations carrying either *DM10* or *DM11* risk alleles. In any case, more definitive proof of the mutual exclusion of *DM10* and *DM11* risk alleles will require more extensive sampling of natural populations across the Iberian peninsula. Co-occurrence of hybrid incompatibility alleles in a single population has been observed before, where different alleles are maintained at intermediate frequencies, but in this case, the hybrids show a milder necrosis phenotype in the lab than Cdm-0×TueScha-9, and no obvious phenotype in the wild (Todesco et al. 2014). The extreme necrotic phenotype caused by the *DM10*–*DM11* interaction, which appears to be largely independent of growth conditions, makes it unlikely that the hybrid phenotype would be suppressed in the wild. In addition, since outcrossing rates of *A. thaliana* in the wild can be substantial (Bomblies et al. 2010), it is conceivable that in some areas these rates are high enough for lethal hybrids to exact a noticeable fitness cost on risk allele carriers.

An interchromosomal relocalization event of the *RLM1* cluster gave rise to *DM10* after *A. thaliana* speciation. Which evolutionary forces might have helped *DM10* to become established on a separate chromosome, if any? NLR genes in clusters are likely to be more mutable than singletons because of illegitimate recombination (Michelmore and Meyers 1998; Baumgarten et al. 2003; Meyers et al. 2005; Wong and Wolfe 2005; Wicker et al. 2007). If *DM10* underwent beneficial neofunctionalization after duplication, its relocation away from the cluster might have stabilized the locus. Another possibility could be conflicts among gene cluster members. Cluster members are sometimes transcriptionally coregulated (Yi and Richards 2007; Deng et al. 2017), so translocation away from the cluster would allow for evolution of new expression patterns for *DM10*. More generally, genomic relocation would enable *DM10* to be subjected to different selection regimes than its cluster homologs. Either way, the fact that the genomic region surrounding *DM10*—different from some other *RLM1* cluster members—is a recombination cold spot (Choi et al. 2016) is consistent with our finding of high LD around the *DM10* locus, especially in accessions carrying the *DM10* risk allele. Together with our phylogenetic results and Tajima’s *D* measurements, this would seem to support the idea of stable *DM10* haplotypes being particularly advantageous.

Although our triple-hybrid cross enabled the identification of the *DM10* and *DM11* QTLs, fine-mapping was complicated by three sets of markers and two loci being involved. Genotyping around the *DM11* locus to differentiate alleles from each of the three grandparents in the mapping cross was further confounded by structural variants, which are typical for NLR-rich regions. Members of the *DM10*-related *RLM1* cluster near the inferred *DM11* QTL are in principle good hybrid necrosis candidates, because TIR domains tend to form homomeric complexes (Zhang, Bernoux, et al. 2017; Dong et al. 2018; Martin et al. 2020); the similar TIR domains between DM10 and RLM1 members make it particularly likely that they oligomerize, which is often an important step in NLR activation. We cannot entirely exclude members of the *RLM1* cluster, because we tested most of them only by coexpression with *DM10* in the heterologous *N. benthamiana* system, and not by genetic inactivation or recapitulation in *A. thaliana*.

In conclusion, we have presented a severe case of hybrid necrosis in *A. thaliana*, where the hybrids show global NLR hyperinduction triggered by the interaction of *DM10*, a relocated singleton NLR gene, and *DM11*, an unlinked locus in chromosome 1. Comparative structure-function analysis of the truncated DM10^TueScha-9^ hybrid necrosis risk allele and the closely related full-length DM10^Col-0^ allele, which does not cause hybrid necrosis, should reveal the exact contributions of LRR and PL subdomains to NLR activity. In addition, the *DM10*/*DM11* case provides a good tool to investigate the consequences of simultaneous activation of a large fraction of NLRs. In the future, by identifying the role of different *DM10* and *RLM1* alleles in response to natural pathogens, one could test whether chromosomal relocation affects how evolution is acting on this group of highly related NLR genes.

## Materials and Methods

Constructs are listed in supplementary table S18, Supplementary Material online, and primers in supplementary table S19, Supplementary Material online.

### Plant Material

Stock numbers of accessions used are listed in Supplementary Material. All plants were stratified in the dark at 4 °C for 4–6 days prior to planting on soil. Late flowering accessions were vernalized 6 weeks under short day conditions (8 h light) at 4 °C. All plants were grown in long days (16 h of light) at 16 or 23 °C at 65% relative humidity under 110–140 μmol m^−2^ s^−1^ light provided by Philips GreenPower TLED modules (Philips Lighting GmbH, Hamburg, Germany).

### RNA Sequencing

Six biological replicates of 10-day-old shoots of Cdm-0×TueScha-9 hybrids and their parental accessions were collected. RNA was extracted as described in Yaffe et al. (2012). The NEBNext magnetic isolation module (New England Biolabs) was used for mRNA enrichment. Sequencing libraries were prepared using NEBNext Ultra II directional RNA library kit and paired-end sequenced (150 bp) in an Illumina HiSeq3000 (Illumina Inc., San Diego) instrument. Reads were mapped against the *A. thaliana* reference TAIR10 using bowtie2 (v2.2.6) (Langmead and Salzberg 2012). Default parameters were chosen unless mentioned otherwise. Transcript abundance was calculated with RSEM (v1.2.31) (Li and Dewey 2011). In silico hybrids were generated to enable midparent value calculations: parental read files were normalized according to sequencing depth and were subsampled by randomly drawing 50% of the reads with seqtk (v2.0-r82-dirty; https://github.com/lh3/seqtk). Differential gene expression analyses were performed using DESeq2 (v1.18.1) (Love et al. 2014). Genes with less than ten counts over all 18 samples were removed from downstream analyses. Significant changes in gene expression between two genotypes were determined by filtering for genes with a |log_2_FoldChange|>1 and *P*adj value <0.01. One read was added to all normalized read counts in figure 1*G* and supplementary figures S2*E* and S6*E*, Supplementary Material online to avoid plotting -INF values in nonexpressed genes (log_10_(0 + 1)=0). Nonadditive gene expression between Cdm-0×TueScha-9 F_1_ hybrids in silico hybrids was analyzed by computing principal components based on the normalized read counts of the top 500 most variable genes across all 18 samples. Plots were generated using the R package ggplot2 (v3.2.0) (Wickham 2009) and heatmaps were plotted using pheatmap (v1.0.8) (Kolde 2012). GO analyses were performed using AgriGO (Tian et al. 2017) using the SEA method. The GO results were visualized with REVIGO treemap (Supek et al. 2011), for clearer visualization only the top 13 and GO categories with the lowest *P* values were plotted in figure 1*G*, the complete list of GO terms is found in supplementary table S2, Supplementary Material online.

### Genotyping-by-Sequencing and QTL Mapping

F_1_ progeny from bidirectional crosses of F_1_ (TueScha-9/Col-0)×(Cdm-0/Col-0) was used as a mapping population. The seedlings showing the hybrid necrosis phenotype versus those that did not, were genotyped individually in a 1:1 ratio. Plants were 10 days old when collected. Genomic DNA was extracted with cetyl trimethyl ammonium bromide buffer (Doyle and Doyle 1987) and then purified through chloroform extraction and isopropanol precipitation (Ashktorab and Cohen 1992). Genotyping-by-sequencing using RAD-seq was used to genotype individuals in the mapping populations with KpnI tags (Rowan et al. 2017). Briefly, libraries were single-end sequenced on a HiSeq 3000 instrument with 150-bp reads. Reads were processed with Stacks (v1.35) (Catchen et al. 2013) and mapped to TAIR10 with bwa-mem (v0.7.15) (Li 2013), variant calling was performed with GATK (v3.5) (McKenna et al. 2010). QTL was performed using R/qtl (Broman et al. 2003) with the information from 348 F_2_ individuals from four independent lines of this segregating population and 6,179 markers.

### De Novo Genome Assembly and Annotation

The Cdm-0 accession (ID 9943; CS76410) was grown as described above. To reduce starch accumulation, 3-week-old plants were put into darkness for 30 h before harvesting. Sixteen grams of flash-frozen leaf tissue were ground in liquid nitrogen and nuclei isolation was performed according to Workman et al. (2019) with the following modifications for *A. thaliana*: eight independent reactions of 2 g each were carried out, and the filtered cellular homogenate was centrifuged at 7,000×g. High-molecular weight DNA was recovered with the Nanobind Plant Nuclei Kit (Circulomics; SKU NB-900-801-01), and needle-sheared 1× (FINE-JECT 26Gx1″ 0.45×25 mm, LOT 14-13651). A 35-kb template library was prepared with the SMRTbell Express Template Preparation Kit 2.0, and size-selected with the BluePippin system according to the manufacturer’s instructions (P/N 101-693-800-01, Pacific Biosciences, CA). In addition, a PCR-free library was prepared with the NxSeq AmpFREE Low DNA Library Kit from Lucigen according to the manufacturer’s instructions (Cat No. 14000-2). The final library was sequenced on a Pacific Biosciences Sequel instrument with Binding Kit 3.0. PacBio long reads were assembled with Canu (v1.71) (Koren et al. 2017). The resulting contigs were first polished using the long reads with the Arrow algorithm (v2.3.2; https://github.com/PacificBiosciences/GenomicConsensus), followed by a second polishing step with PCR-free short reads using the Pilon algorithm (v1.22) (Walker et al. 2014). Lastly, the resulting contigs were scaffolded based on TAIR10 assembly by REVEAL (v0.2.1) (Linthorst et al. 2015). The previously generated Cdm-0 transcriptome sequencing data were mapped against the scaffolded genome assembly using HISAT (v2.0.5) (Kim et al. 2015). Subsequently, the mapping results were used as extrinsic RNA sequencing evidence when annotating the genome using AUGUSTUS (v3.2.3) (Stanke et al. 2006). Transposable elements and repetitive regions were identified and masked prior to gene annotation using RepeatModeler2 (v2.01) (Flynn et al. 2020). Orthologous genes shared between Cdm-0 and the current *A. thaliana* reference annotation from Araport11 were identified using Orthofinder (v2.4.0) (Emms and Kelly 2019).

### Manual NLR Annotation of the *DM11* Mapping Interval

The 20- to 25-Mb region of chromosome 1 was extracted from the Cdm-0 assembly. The assembly was used as a query against a subject FASTA file containing 167 NLR genes from the Col-0 reference accession using BlastN (Altschul et al. 1990). Hits were binned in 20-kb intervals and the percentage identity between the queries and the subject was visualized across all bins. NLRs between At1g56510 to At1g64070 in Col-0 found in this interval were manually annotated based on the percentage identity plotted and on AUGUSTUS gene predictions (v2.5.5) (Stanke et al. 2006).

### Genome-Wide Association Study

Cdm-0-dependent hybrid necrosis in the F_1_ progeny from crosses with 80 accessions (Chae et al. 2014) was scored as 0 or 1. The binary trait with accession information was submitted to the easyGWAS platform (Grimm et al. 2017) using the FaSTLMM algorithm. A −log_10_(*P* value) was calculated for every SNP along the five *A. thaliana* chromosomes.

### Constructs and Transgenic Lines

Genomic fragments were PCR amplified, cloned into pGEM-T Easy (Promega, Madison, WI), and then transferred to the binary vectors pMLBart, pCambia1300, or pFK210. Constructs were introduced into plants using *Agrobacterium*-mediated transformation (Weigel and Glazebrook 2002). T_1_ transformants were selected on BASTA (pMLBart and pFK210) and crossed to incompatible accessions. Ethanol-inducible constructs were PCR amplified, cloned into pGEM-T Easy, as part of a separate experiment, 2xHA tags were added via PCR and the whole fragment, which was then transferred to the pCR8 entry vector (ThermoFisher Scientific). Next, the genomic fragment was moved to the destination vector pZZ006 (Caddick et al. 1998) through the Gateway LR reaction (ThermoFisher Scientific). Quality control for all constructs was done by Sanger sequencing. For transient expression in *N. benthamiana*, *A. tumefaciens* strains ASE (*RLM1*) or GV3101 (*DM10* and At1g57650) were grown to an OD_600_ of 1.2–1.8 and incubated in induction medium (10 mM MES [pH 5.6], 10 mM MgCl2, and 150 μM acetosyringone) overnight. The cell suspensions were normalized to an OD_600_ of 0.8 and coinfiltrations suspensions were mixed 1:1. Suspensions were then infiltrated into the abaxial side of *N. benthamiana* leaves. In the case of EtOH-inducible constructs, infiltrated *N. benthamiana* was induced at 18-h postinfiltration by irrigation with 1% ethanol and kept within a transparent plastic dome for another 18 h. *DM10 N. benthamiana* constructs shown in figure 4 were coexpressed with a *35S::GFP* construct as part of a larger experiment to test for candidate *DM11* loci.

### Population Genetic Analyses

Amino acid sequence conservation scores were calculated with ConSurf (Armon et al. 2001; Ashkenazy et al. 2016). SNPs occurring in repetitive regions and only present in one of the 73 extracted *DM10* alleles were considered sequencing errors and were manually curated. Protein domains were predicted using InterProScan (Jones et al. 2014). LRR domains were predicted with LRRsearch and the score threshold was set at 7 (Bej et al. 2014). NLR motifs were defined based on previous studies (Meyers et al. 2003; Shao et al. 2016). Nonsynonymous to synonymous substitution rates were calculated using KaKs_Calculator (v2.0) (Wang et al. 2010) with the NG method (Nei and Gojobori 1986); a window length of 60 bp and a step size of 6 bp were chosen. Genomic regions of interest were subsetted from a 1,135 genomes VCF file (1001 Genomes Consortium 2016) using VCFtools (v0.1.14) (Danecek et al. 2011). The resulting VCF file was filtered by MAF = 0.01 and a maximum percent of missing data per SNP of 30%. Sequences were converted to FASTA, aligned with MUSCLE (v3.8.31) (Edgar 2004), and then visualized with Aliview (v1.18.1) (Larsson 2014). NJ trees were calculated with Fastphylo (v1.0.1) (Khan et al. 2013) and visualized with iTol (Letunic and Bork 2007) (https://itol.embl.de/shared/cbarragan). Maximum-likelihood trees were calculated with RaxML (v0.6.0) using the GTR+G4 model (Stamatakis 2014). Linkage disequilibrium (*r*^2^), was calculated with PLINK (v1.90) (Purcell et al. 2007). PCAs were calculated with smartPCA (Patterson et al. 2006). Tajima’s *D*, *F*_ST_, and nucleotide diversity (*π*) were also calculated with VCFtools. Maps were created with the R-packages maps (v3.3) and ggmap (v3.0) (Kahle and Wickham 2013). Admixture groups were assigned to each accession in accordance with the 1001 Genomes project (1001 Genomes Consortium 2016); since TueScha-9 had not been part of that study, admixture group assignments for it were estimated based on the genetic make-up of neighboring accessions. *RLM1* homologs in *A. lyrata* and *B. rapa* were identified using the Ensembl Plants portal (Bolser et al. 2016). Sequences from the genome assemblies TAIR10 (*A. thaliana*), *B. rapa* (v1.5), and *A. lyrata* (v1.0) were used for phylogenetic analyses.

## Supplementary Material

Supplementary data are available at *Molecular Biology and Evolution* online.

## Supplementary Material

msaa245_Supplementary_Data
